# AI-Driven Security: Detecting Cyber Attacks in IoT Networks

**DOI:** 10.3390/s26175321

**Published:** 2026-08-22

**Authors:** Jawad Hussain Awan, Misbah Safdar, Muhammad Ayaz Shirazi, Min Young Kim

**Affiliations:** 1 Faculty of Engineering, Sciences and Technology, Iqra University, Main Campus, Karachi 75550, Pakistan; 2 School of Electronic and Electrical Engineering, Kyungpook National University, Daegu 41566, Republic of Korea; 3 Research Center for Neurosurgical Robotic Systems, Kyungpook National University, Daegu 41566, Republic of Korea; 4 KNU-LG Electronics Convergence Research Center, Kyungpook National University, Daegu 41566, Republic of Korea

**Keywords:** cyberattack detection, deep learning, intrusion detection system and analysis

## Abstract

Traditional rule-based intrusion detection systems generally fail in identifying unknown or evolving threats; thus, automated and adaptive kinds of methods are crucial. Deep learning models provide promising solutions, but many recent studies depend on hybrid architecture, which increase the computational cost and reduce deploying ability on real-time or resource-limited systems. In this paper, we present and test a standalone LSTM model for multiclass cyberattack detection based on a CIC_IoT_Dataset2023, a recent labeled dataset that mirrors the actual network environment containing 33 attack categories. The dataset was extremely imbalanced as benign traffic accounted for most of the classes. To detect such attacks, we used the Synthetic Minority Oversampling Technique (SMOTE) to increase the frequency of less common types of address. The pre-processed dataset was then employed to train four models (RNN, CNN, DNN and the proposed LSTM) for performance analysis with sequential data. The proposed LSTM model achieved an accuracy between 2% and 7%. LSTM had good detection for frequent attacks and slow-changing patterns, which shows its capacity in learning long-lasting dependencies. The results demonstrate that a simple, lightweight standalone LSTM model can be used for effective and realistic intrusion detection without the need for complex hybrid architecture.

## 1. Introduction

The Internet of Things (IoT) has introduced a paradigm shift to the digital communication world by interconnecting billions of devices spread over homes, infrastructure and healthcare systems [1,2]. These linked systems produce huge amounts of data and real-time automation, but they also have created new cybersecurity threats. Due to the restricted computing capacity and insufficient security setting for most IoT devices, they become vulnerable to attackers [3]. These systems have come to play a major role in society, and cyberattacks affecting these systems, such as data breaches, tampering of devices, and service interruptions, are increasingly severe and widespread [4,5]. With this ever-changing surface area, the need for efficient, scalable and accurate intrusion detection systems (IDS) to secure modern networks has become more pressing than ever.

Signature-based and rule-based detection mechanisms are the traditional IDS models. Such systems can sufficiently detect previously seen types of attacks; however, they are weak when it comes to detecting new or altered (zero-day) attack patterns [6,7]. It is common for attackers to adapt their attacks, and static security policies are not sufficient to deal with the dynamic nature of attacks [8]. The traditional IDS techniques become ineffective and difficult to manage as the traffic in the network grows and becomes complex [9]. To overcome these limitations, researchers have moved towards AI and machine learning (ML) methods that automatically learn how networks work [10,11].

Deep learning models have shown state-of-the-art performance in detecting anomalies and cyberattacks on network traffic [11,12]. Recurrent neural networks (RNNs) and Long Short-Term Memory (LSTM) networks are popular among such models due to their ability to learn from sequential data. Network traffic follows time-based temporal behavior. Thus, LSTM is a relevant model for this context [13,14]. Research has shown that LSTM is able to learn long-range dependencies and can also detect some quiet but slight attempts at evasion or probing from scans, such as slow-scale or increasing request frequencies [15,16]. This assists in maintaining the memory of historical data and helps it perform better than traditional vanilla RNNs, which tend to suffer from problems such as vanishing gradients [17].

The idea to utilize LSTM in cyber-security was also based on the supporting evidence identified in recent findings. Some research works have proved that LSTM-based IDS models reach a high accuracy for detecting DDoS, brute-force, command injection, and botnet attacks [18,19,20,21,22]. Other related works also described the advantages of utilizing LSTM in predicting workloads, monitoring cloud systems, and modeling dynamic behavior in large networks [23,24,25]. Their best model achieved a validation loss of 0.0045, indicating strong convergence during training. CNNs are good at extracting spatial features but are bad at temporal relations. DNNs cannot model long term-dependent relationships, and they are less effective for time series traffic data [26,27,28,29].

Furthermore, hybrid models like CNN–LSTM and the ensemble of RNN architecture have also been widely studied in the literature [30,31,32]. These models incorporate both spatial and temporal feature learning and, in many cases, achieve high detection accuracy. Nevertheless, their high training time and computation cost and difficulty in deployment (especially within the context of resource-constrained IoT settings) are drawbacks. Consequently, some studies suggest that a trained solo LSTM can perform equally as effectively as the best deep models and keep the process simple and neat [33,34].

These are the challenges that lead us to consider a deep learning model specially designed for network traffic patterns in practice. This study evaluates the capability of a single LSTM model trained only on the state-of-the-art CIC-IoT-Dataset2023 containing realistic IoT experiences, multiple attack types, and recent threats. By utilizing proper pre-processing and class-balancing schemes, this work assesses if a simple LSTM can obtain high detection accuracy without relying on complicated hybrid architecture.

The goal of this work is to bridge a gap in the literature by answering how a single LSTM model works on current IoT datasets and if it can be used as an efficient, lightweight, and deployable option for real-time cybersecurity systems. The results provide useful insights in the quest to build efficient IDS models that will be able to cope with sophisticated cyber threats as we continue to move into an era of ubiquitous connectivity. The novelty of this work is not in proposing a new LSTM architecture. Instead, it offers a systematic and reproducible comparison of four widely used deep learning models as part of a uniform preprocessing, training, and evaluation process applied to the recently released CIC_IoT_Dataset2023. Despite this, comparisons such as these are still useful since there are many studies that are available using different datasets with different engineered features, different preprocessing techniques, and different evaluation protocols, making direct comparisons difficult. A baseline stand-alone LSTM architecture is explored, which is easily implementable in the IoT context. The proposed single-layer LSTM reduces the number of trainable parameters and computation complexity compared with the deeper hybrid architecture reported in the literature. Thus, it can be considered computationally simpler, but not necessarily as lightweight as possible. The issues of resource-constrained hardware deployment were not addressed and will be explored in future work.

## 2. Literature Review

Ongoing research has focused on the potential benefits of combining multiple deep learning architecture for capturing spatial as well as temporal features. As an illustration, Kaur and Singh built a hybrid deep recurrent network that increases the performance of intrusion detection as compared to traditional RNN models [35]. In the same way, ensemble-based strategies with the use of stacked or parallel deep neural architecture have achieved high detection accuracy in different kinds of traffic environments [36]. Shurman provided evidence of deep learning technique capability in recognizing the occurrence of both DoS and DDoS by means of automated feature extraction; thus, deep learning methods were mostly able to supersede the performance of the previous IDS solutions [37]. Furthermore, hybrid IDS architecture targeted at IoT networks have been able to raise the level of the network, based on research by Smys, which combines multiple detection components to resolve the issues of IoT-specific vulnerabilities [38].

In a similar way, Ref. [39] used a hybrid of CNN, Self-Organizing Maps (SOMs), and Naive Bayes to detect cyberattacks, with the main issues being extreme imbalance in the classes and a high false-positive rate, especially with DoS attacks. An overview of host-based IDSs was conducted in Ref. [40], in which deep-learning models were evaluated (LSTM, CNN, and GRU) on the detection of anomalies on the basis of system calls; the performance of the models was better, including higher rates of detection, but they also had high ratios of false-positives and the inability to detect zero-day attacks. To deal with threats of IoT, Ref. [41] proposed AS-IDS, a hybrid anomaly- and signature-based IDS based on deep learning and reinforcement learning, and reported a detection rate of 96.9%, where false alarms were low compared to the traditional DBN and RNN methods. A CNN-based self-normalizing deep neural network (DNN) was suggested in Ref. [42] to address the issue of class imbalance in IoT intrusion datasets; it reached an accuracy of 99.95% and markedly enhanced the detection of minority classes using sophisticated data augmentation methodology, including CTGAN. Moreover, the STL-HDL platform in Ref. [43] used hybrid CNN–LSTM models with sampling mechanisms to address imbalanced big data conditions to achieve more than 99% accuracy in CIDDS-001 and UNSW-NB15 datasets. Lastly, Ref. [44] devoted itself to wireless sensor networks and offered a DNN-based IDS with cross-correlation feature selection, outperforming the conventional machine learning algorithms; however, the question of scalability and resilience to large-scale implementations is still a research question.

Other studies focus on the reliance of deep learning models like convolutional neural networks (CNNs) and gated recurrent mechanisms for network traffic data processing. Khan came up with a Hybrid Convolutional Recurrent Network (HCRNN) that uses CNNs for spatial feature extraction and RNNs for sequence learning of interaction detection accuracy [45]. Similarly, they proved that deep sequential models could be instrumental in depicting behavior changes in IoT servers, thus pointing to the importance of recurrent architecture for the capture of attack signatures, which are evolving [46].

LSTM-based models additionally prolong the benefits of these models by solving the issue of disappearing gradients, being able to remember long-term relationships in traffic flows, and thus being applicable to both rapidly changing and slowly evolving attack classes [47].

Since real datasets are characterized by a severe class imbalance situation in which some attacks are very rare, several authors have decided to use oversampling, feature-level correction, or dynamic model training strategies. Liu et al. have proven that deep learning models can substantially enhance their performance if they are trained on datasets that have been corrected by means of oversampling and feature-based balancing [48]. Ashiku and Dagli went on to support this statement by proving that deep neural networks have become more stable and reliable when trained on enriched and balanced intrusion datasets [49]. The use of bidirectional LSTM architecture has equally dealt with temporal imbalance, and they have demonstrated strong capability in detecting rare attack sequences [50].

Recent studies are primarily concerned with the creation of efficient IDS for cloud networks, the Internet of Things, and software-defined networking (SDN) systems. DDoSNet, a deep learning technique in a cloud environment, was introduced by Srilatha and Thillaiarasu to respond to high-volume attack traffic effectively [51]. Deep learning-powered SDN-based IDS architecture also have a bright future, as they provide not only centralized control but also dynamic traffic monitoring, which was proven in research by Chaganti. These mechanisms are empowered by LSTM and CNN structures, through which they can recognize intrusions instantly and self-modulate network traffic. In the same way, Halbouni et al. pointed out the advantages of the CNN–LSTM combined framework in the case of IoT devices, achieving the improvement of both accuracy and speed of execution [52].

Initially, research turned to the importance of interpretability in models and their lightweight design. Qazi and his team came up with an IoT IDS model architecture that could run with minimal energy consumption while keeping its accuracy at a high level [53]. Ravi and his coworkers put forward a recurrent ensemble method as a feature fusion technique, and thereby their deep learning models might seamlessly integrate multi-layered temporal information for robust detection performance [54]. Hnamte and Hussain’s dual-stage DCNN-BiLSTM hybrid model focuses on temporal-spatial feature extraction for intrusion detection with a very high degree of accuracy. In their research, they demonstrated the significance of linking CNN and LSTM components together [55].

Experimentation with strictly signature-based and anomaly-based intrusion detection systems has also attracted attention. Moorthy studied and revealed the signature elements of hybrid IDS models, thereby upgrading their competence in detecting both previously known and new types of attacks [56]. Awajan presented a deep learning-based IDS only for IoT networks, which can learn device-level behavior changes and become compatible with heterogeneous traffic patterns [57]. There have also been proposals for lightweight IDS systems that can function in environments with severe computational limitations. Wang et al. researched dynamic quantization methods for IoT environments to compress deep learning models without causing a reduction in accuracy [58]. Further work by Wang et al. introduced a lightweight BERT-of-Theseus-based detection model, thus illustrating how transformer architecture can be efficiently utilized for intrusion detection purposes [59].

LSTM architecture are still a vibrant option and continue to be highly effective in real-time detection tasks. Research to that effect also shows that LSTM not only is able to handle long temporal dependencies but also to adjust sequence variable-lengths that come from real traffic data [60].

Further, the research on anomaly-based IDS has led to the conclusion that IoT systems are the beneficiaries of deep learning architecture that can detect the occurrence of irregular activity by first modeling normal behavior patterns [61].

Chaganti has shown that SDN-enabled IoT networks are also heavily influenced by LSTM-based methods in a positive way. In their study, the LSTM models exhibited a robust detection performance for a variety of attack types [62]. Lately, research also aligns with the argument of hybrid LSTM-based solutions as the best choice to unpack spatial–temporal features with high-accuracy detection results [63]. The necessity of advanced detection mechanisms is further being realized in works that investigate the combinations of fuzzy logic and deep learning as the most potent and intelligent IDS frameworks that are adaptable and can be easily deployed in the real world.

In general, earlier studies have been quite explicit about the fact that deep learning, especially LSTM and hybrid sequential models, has been a major factor in the protection of contemporary IoT and network environments. Although hybrid methods usually provide high accuracy, their complicated nature makes it difficult to deploy them. Therefore, a higher proportion of recent articles is devoted to the advantage of a single LSTM model that achieves a good compromise between accuracy, efficiency, and adaptability in real time. The primary aim of this current research is to examine LSTM-based intrusion detection with the help and agreement of the latest IoT-oriented CIC_IoT_Dataset2023.

## 3. Research Methodology

This study followed a structured methodology to develop and evaluate an LSTM-based intrusion detection system using the CIC_IoT_Dataset2023. The research process consisted of four main stages: dataset preparation, data preprocessing, model design, and performance evaluation.

### 3.1. Dataset Description

This research is based on the CIC_IoT_Dataset2023 created by the Canadian Institute for Cybersecurity (CIC), University of New Brunswick. The dataset was produced in a real IoT environment with 105 different devices, such as smart home, industrial, and consumer IoT devices. It can capture over 46 million network flow records with labels for benign and malicious traffic that can serve as a comprehensive reference to test IoT intrusion detection methods on a large scale. There are 33 attack types throughout the dataset, which are divided into seven larger attack groups: DDoS, DoS, Reconnaissance, Web-based attacks, Brute Force, Spoofing and Mirai. These attacks were performed by malicious IoT devices against other IoT devices, allowing for the realistic assessment of IoT security analytics models.

The attacks in the DDoS category encompass ACK Fragmentation, HTTP Flood, ICMP Flood, ICMP Fragmentation, PSHACK Flood, RSTFIN Flood, SYN Flood, SlowLoris, Synonymous IP Flood, TCP Flood, UDP Flood, and UDP Fragmentation. DoS (Denial of Service) attacks include HTTP Flood, SYN Flood, TCP Flood and UDP Flood attacks. Host Discovery, OS Scan, Ping Sweep, Port Scan, and Vulnerability Scan attacks are part of the Reconnaissance category attacks. Browser Hijacking, Command Injection, SQL Injection, Uploading Attack, XSS, and Backdoor Malware are the types of attacks that are included in the Web-based category. The Brute Force category contains Dictionary Brute Force, and the Spoofing category contains DNS Spoofing and MITM ARP Spoofing attacks. Greeth Flood, GreIP Flood and UDPPlain attacks are included in the Mirai category.

The CIC_IoT_Dataset2023 uses statistical traffic features extracted from packet capture files to represent each network flow. It includes 45–46 features of numeric values extracted from flow-level data descriptions of temporal, statistical and protocol-level characteristics of a network communication. These features are flow duration, header length, protocol type, packet rate, source and destination traffic rates, TCP flag statistics, inter-arrival time (IAT), packet size statistics, magnitude, radius, covariance, total packet size and total packet count. The flow-level characteristics were used as input features for deep learning model training and evaluation in this study. Table 1 also illustrates each attack category along with details.

### 3.2. Data Preparation

During preprocessing, categorical labels were encoded into numerical values. For example, attack labels such as “DNS_Spoofing” and “CommandInjection” were transformed into integer class identifiers. Features with missing values were imputed using column means. After normalization, all feature values were scaled into the [0, 1] range using Min–Max scaling. Additionally, a multi-step preprocessing pipeline was put together to make sure that the data were of good quality and that the model performance was high.

(1) Data Cleaning:

All non-numeric attributes were either removed or converted. Missing values were replaced with the mean values of the columns. Duplicate records were checked, and if there were any, they were removed so that the model would not be biased.

(2) Feature Scaling:

As features had different numeric ranges, Min–Max normalization was used to scale all the values between 0 and 1. This facilitates training to be more stable, and the large-value features do not get to dominate gradient updates.

(3) Handling Class Imbalance (SMOTE):

As some attack categories had a very small number of samples compared to others, the Synthetic Minority Oversampling Technique (SMOTE) was employed to create artificially but statistically consistent samples for the minority classes. This made sure that the model did not get biased toward the most frequent attack types, and therefore, its ability to detect rare or low-frequency attacks was enhanced.

(4) Data Splitting:

The dataset was first divided into training (80%) and testing (20%) subsets using stratified sampling. During the avoidance of data leakage, SMOTE was applied only to the training dataset. The test dataset remained unchanged and retained the original class distribution for unbiased evaluation. The division ensured that both sets contained all the categories of attacks—common and rare.

In Table 2, the results indicate that SMOTE improved minority attack detection performance across all models. The largest improvement was observed in recall values for underrepresented attack classes.

Table 3 presents the class-wise effect of SMOTE on selected CIC_IoT_Dataset2023 classes where valid F1-score values were available before and after oversampling. The results indicate that SMOTE provides the largest improvements for minority attack categories, particularly Uploading Attack (+26.23%), SQL Injection (+21.54%), XSS (+19.12%), and Mirai-UDPPlain (+16.67%). In contrast, the benign class shows no change after applying SMOTE, indicating that oversampling mainly benefits underrepresented attack categories rather than majority classes. These findings demonstrate that SMOTE improves minority-class representation and enhances the model’s ability to detect less frequent attack behaviors.

### 3.3. LSTM Model Architecture

A standalone Long Short-Term Memory (LSTM) model was chosen primarily because of its capability to learn sequential patterns in network traffic. LSTM networks are a type of neural network architecture to solve the vanishing gradient problem and remember long-term dependencies; hence, they are the best fit for time-series cyberattack detection.

In Table 4, a unidirectional LSTM layer with 128 hidden units was employed. Bidirectional LSTM was not used in this study to maintain a lightweight architecture suitable for deployment in resource-constrained IoT environments. The model architecture utilized in this study comprised the following:

- Input Layer: The layer that takes in the normalized numerical features.
- LSTM Layer: The main hidden layer that can learn the time-based and sequential behavior of network traffic. This layer captures not only short but also long temporal dependencies.
- Dense (Fully Connected) Layer: The layer that compresses the learned representations into more understandable features for classification.
- Output Layer: A SoftMax-based multi-class classifier that labels each sample with one of the attack or normal categories.

To ward off overfitting and to enhance the model’s generalization capability, a dropout mechanism was introduced. The model was compiled with the Adam optimizer and categorical cross-entropy was used as the loss function.

In this work, the goal is to compare the representative models with the standalone deep learning models in the same experimental settings and not against highly optimized hybrid architecture. In recent years, CNN–LSTM, BiLSTM, and attention-based models have generally reported better performance; however, most of these models have greater complexity in computation and different preprocessing pipelines that make it difficult to directly compare the models.

### 3.4. Model Training Setup

The model was trained on the balanced dataset created by SMOTE to make sure that the model learned equally from all attack categories. The training parameters were as follows:

- Epochs: 20.
- Batch Size: 64.
- Optimizer: Adam.
- Loss Function: Categorical Cross-Entropy.
- Evaluation Metrics: Accuracy, Precision, Recall, and F1-Score.

The training was performed with Python and TensorFlow on Google Colab. Early stopping was used to stop overfitting and to halt the training process automatically when the validation loss stopped improving.

### 3.5. Performance Evaluation

To evaluate the efficiency of the model in recognizing various attacks, global and local class-wise evaluation methods were employed.

(1) Standard Metrics:

The model’s overall detection capability was verified by computing accuracy, precision, recall, and F1-score. These metrics are usually implemented in IDS studies as they open the way to the understanding of false-positives, false-negatives, and classification reliability.

(2) Confusion Matrix:

The LSTM’s attack category identification extent was represented by means of a multi-class confusion matrix. On the diagonal, the matrix pointed out the correctly classified samples, and in the off-diagonal cells, it displayed the misclassifications. The stage was instrumental in identifying attacks that the model could easily or hardly distinguish.

(3) Comparative Analysis:

The LSTM model’s work was put side by side with that of three other deep learning models—RNN, CNN, and DNN—to decide which was better. The LSTM was the best one because, although all the models used the same dataset, LSTM could handle time-based dependencies in traffic sequences.

### 3.6. Research Workflow Summary

- The entire methodology can be summarized in the following steps:
- Load CIC_IoT_Dataset2023.
- Clean and normalize the features.
- Balance dataset using SMOTE.
- Split into training and testing sets.
- Build an LSTM-based intrusion detection model.
- Train using optimized parameters and early stopping.
- Evaluate performance with metrics and confusion matrices.
- Compare results with RNN, CNN, and DNN.

**Figure 1:**
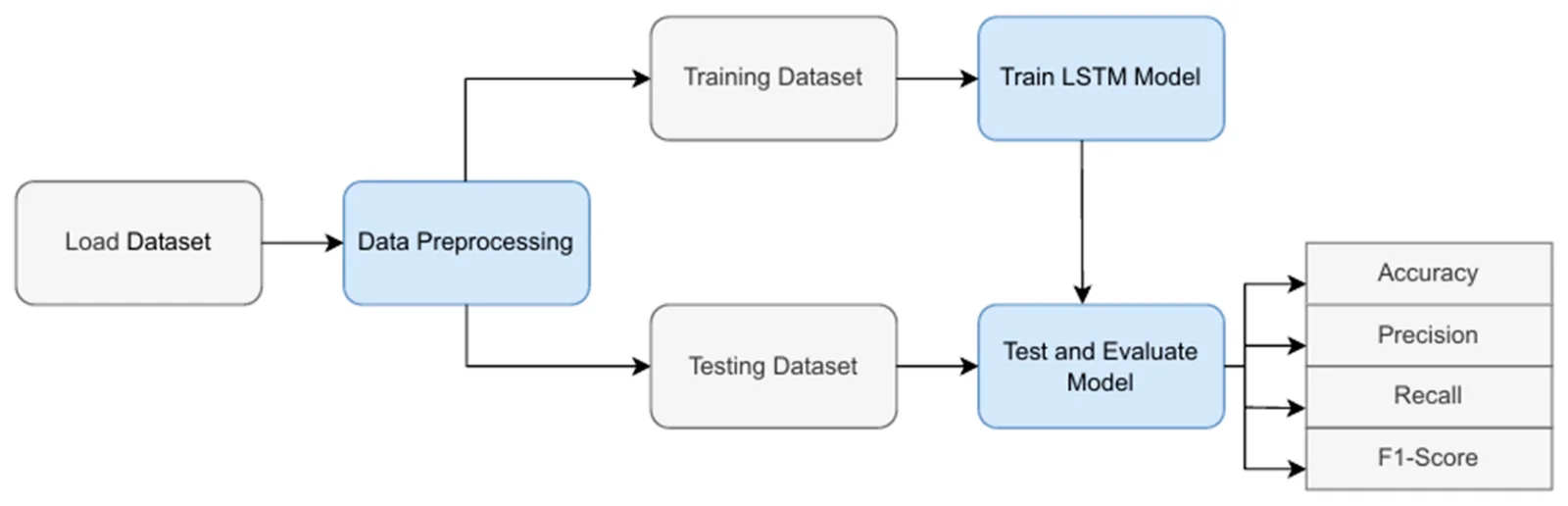
LSTM model training and evaluation process.

This systematic flow, as depicted in Figure 1, guarantees the reliability, repeatability and compliance of the results to meet the intrusion detection needs of the IoT networks in practice.

## 4. Results

Several deep learning models are presented and analyzed on the CIC_IoT_Dataset2023 for cyberattack detection. The main focus is to compare the performance of the LSTM model with other models, such as RNN, CNN and DNN. All the models were trained with time-series data of network traffic, and the network data were pre-processed with common techniques like class balance (SMOTE). We evaluated the models using widely used performance metrics such as accuracy, precision, recall, and F1-score.

### 4.1. Modeling and Results Interpretation

#### 4.1.1. RNN

**Figure 2:**
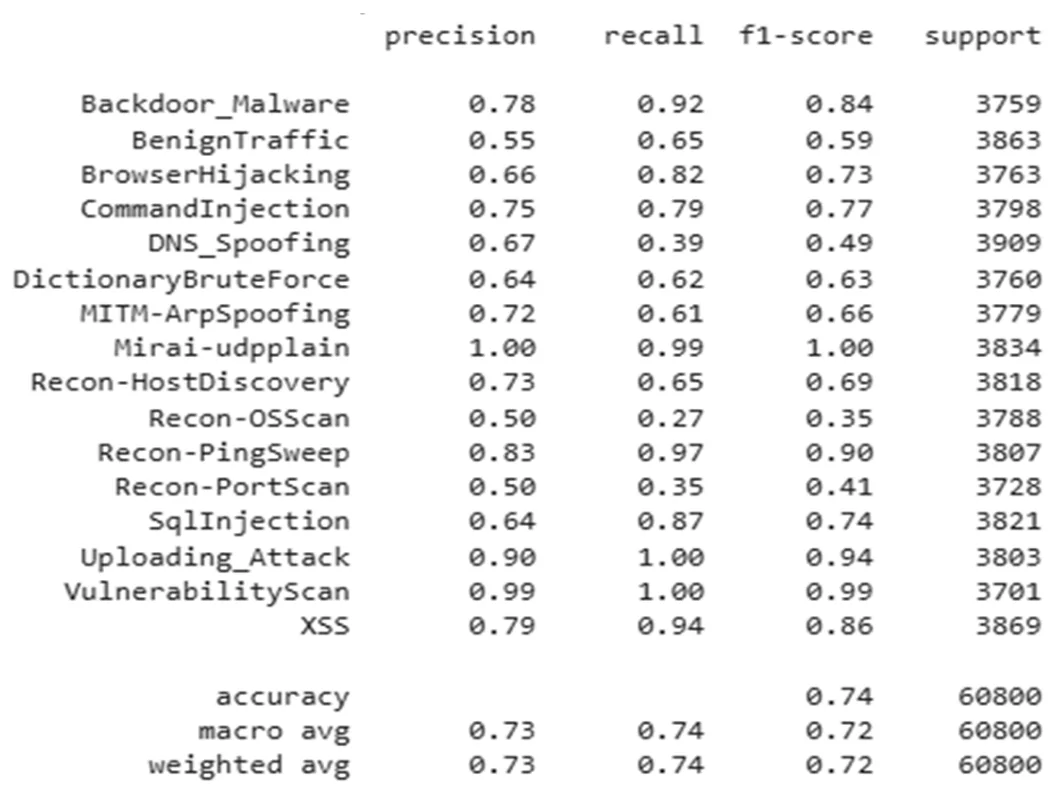
Evaluation metrics for RNN.

The accuracy of the RNN model in Figure 2 was 73%. It appears to be accurate in forecasting most attacks. Attacks that were easily detected were Mirai-UDPPlain and Uploading_Attack for recognition and recall, but Recon-PortScan was difficult to discover. The value of precision was 0.72, the recall value was 0.73, and the F1-score was 0.71, indicating fair performance with complexity issues on multi-class recognition; simple patterns were found to be effective on RNN but they failed to perform on rare or more complex attacks.

**Figure 3:**
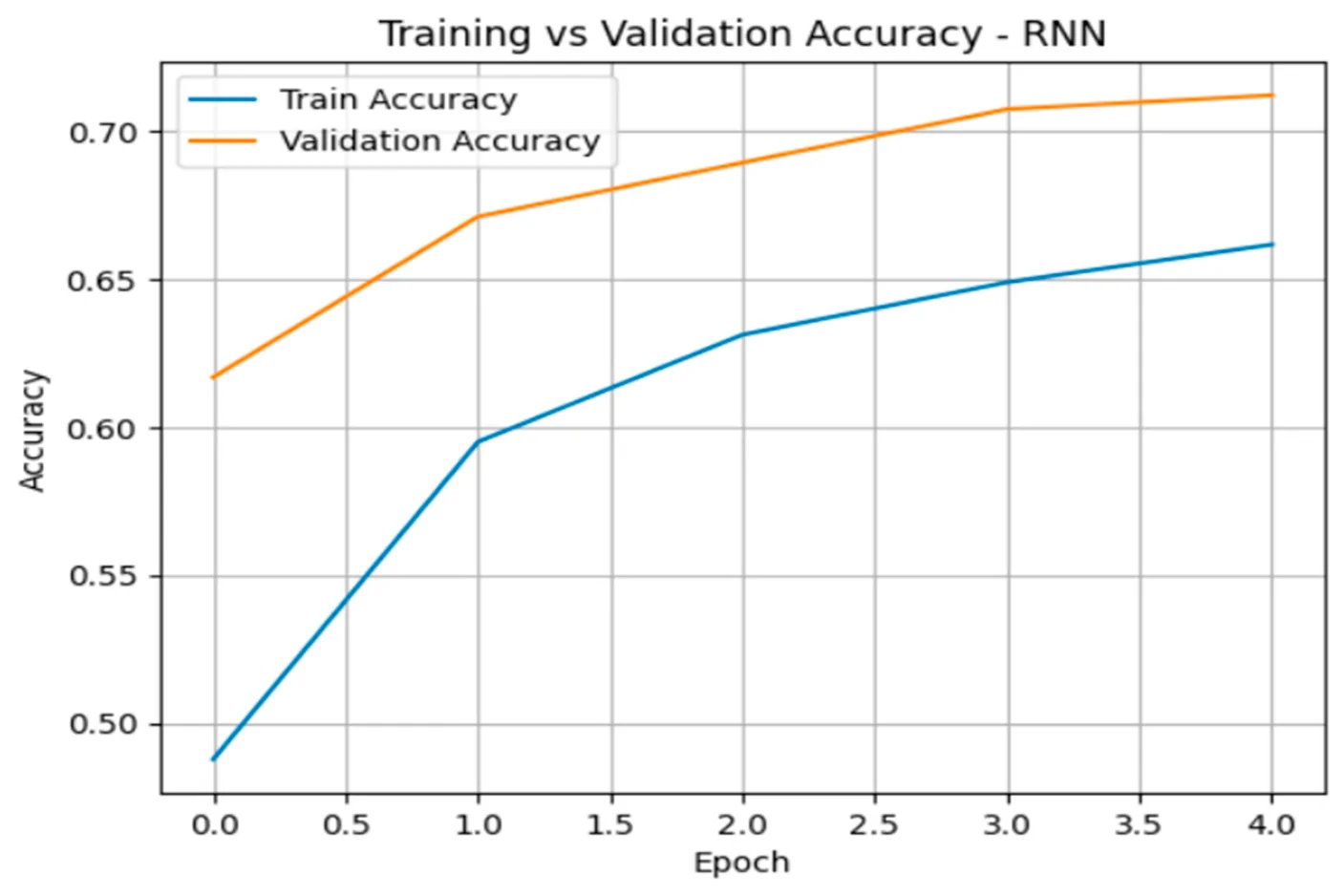
RNN accuracy plot.

The accuracy rate of the RNN model was plotted in Figure 3, which was 73%. RNNs can deal with sequences, but they have the problem of the vanishing gradient, in which it is difficult to keep long-term information. Consequently, their performance was less effective compared to LSTM, particularly for detecting long-duration attacks.

**Figure 4:**
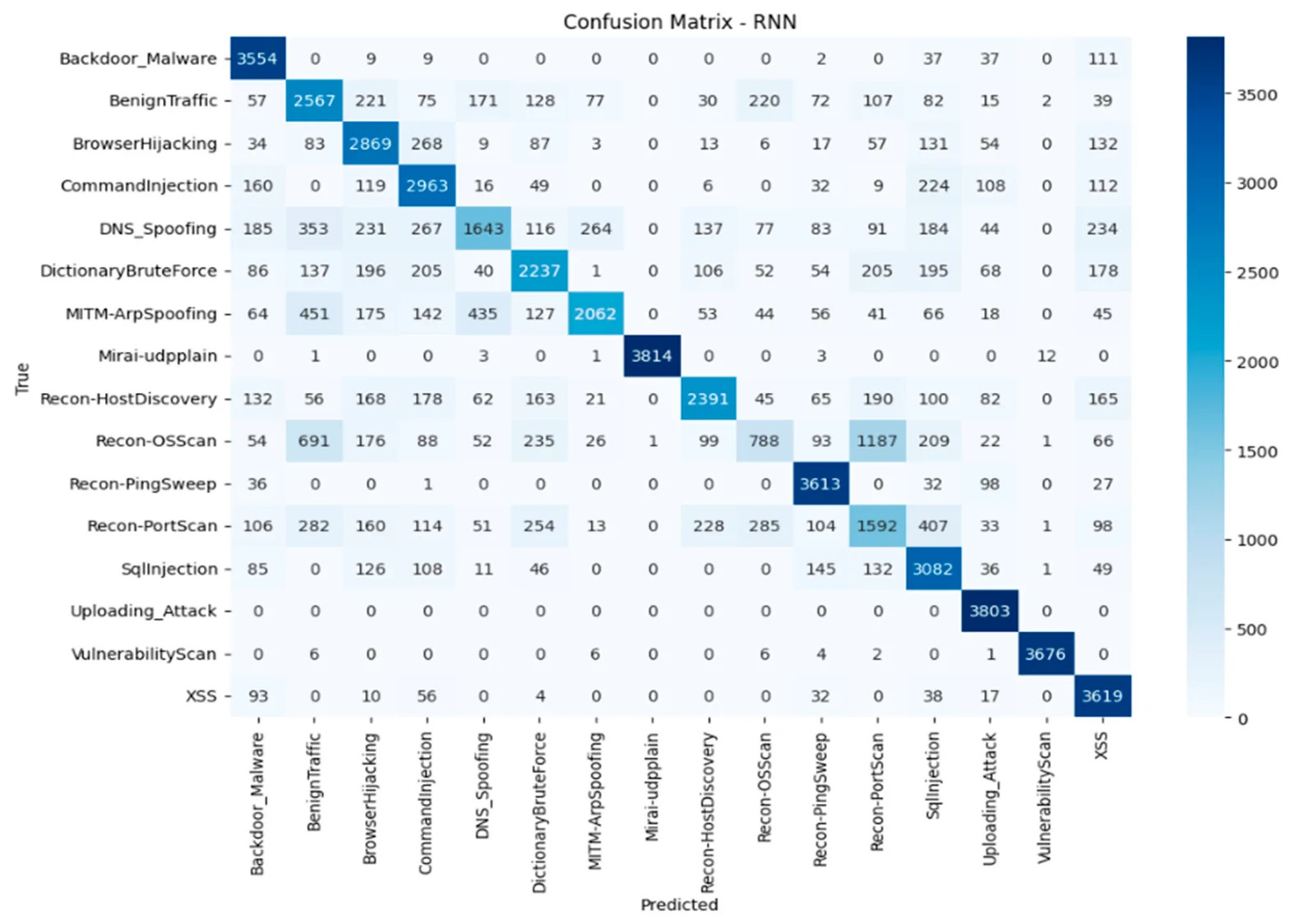
RNN confusion matrix.

Figure 4 illustrates the confusion matrix, showing how the RNN model predicted each type of attack and normal traffic. The model’s prediction is displayed in each column, and the actual class is displayed in each row. Darker boxes represent a greater value or more accurate prediction. We can observe from the matrix that the RNN model was able to identify a lot of attacks, such as Mirai-UDPPlain, Uploading_Attack, and XSS, which are given a high value on the diagonal. In one instance, 3814 samples of Mirai-UDPPlain were correctly classified, and 3619 XSS attacks were also identified correctly. However, it has a couple of misclassifications. In this case, the model failed to distinguish between BrowserHijacking, BenignTraffic, DictionaryBruteForce and DNS_Spoofing. The overlapping predictions suggest that the RNN model confuses similar attack types or has trouble with noisy data some of the time. This is one of the recognized weaknesses of RNNs, that they do not retain information over time, affecting their ability to identify complex and similar patterns over time. They can accurately identify some attacks but less accurately than other models like LSTM.

#### 4.1.2. CNN

**Figure 5:**
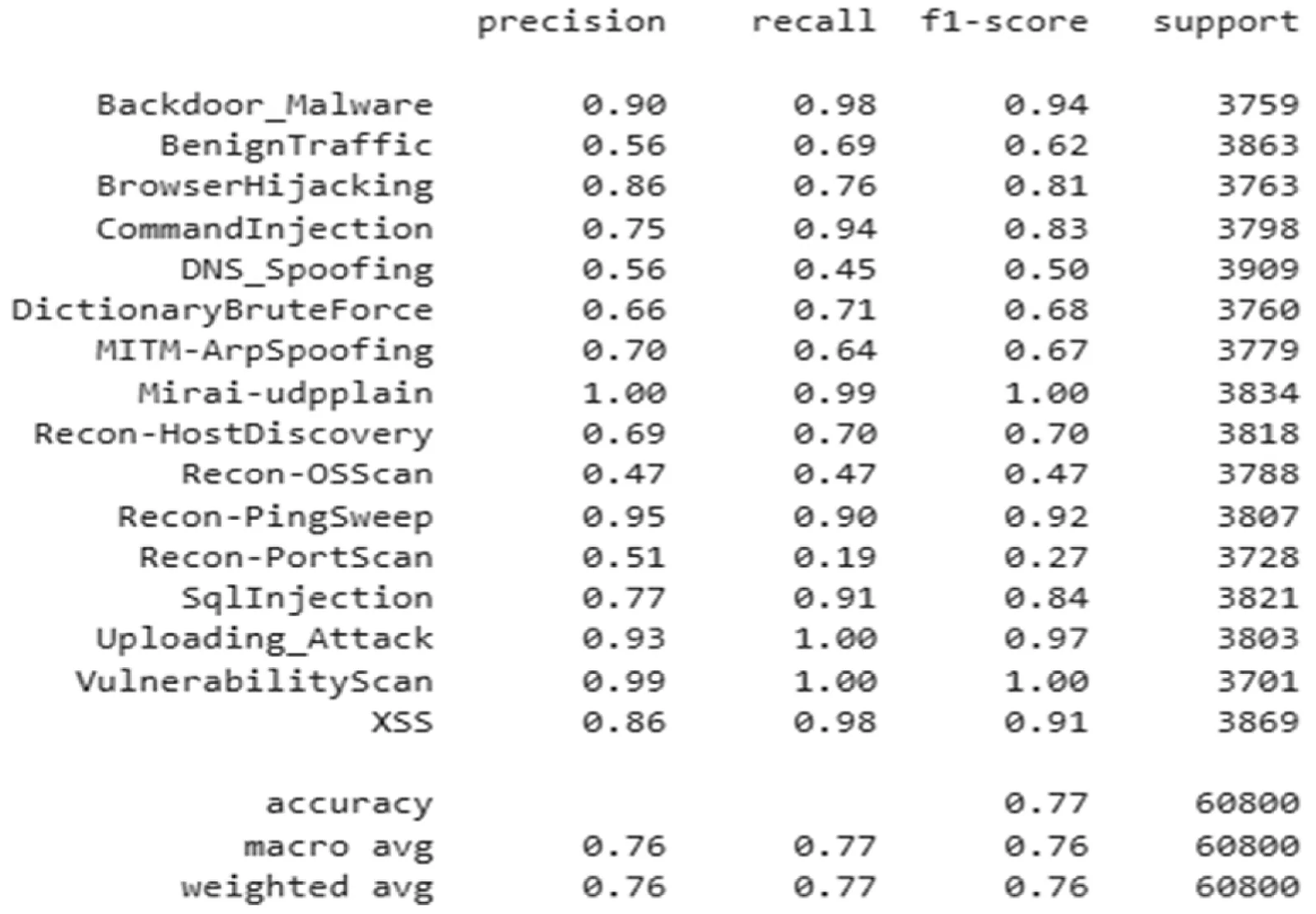
Evaluation metrics for CNN.

The CNN model was able to achieve an accuracy level of 78% and performed particularly well for attacks like Mirai-UDPPlain, Uploading_Attack, and VulnerabilityScan, as shown in Figure 5. Good results were also obtained for CommandInjection and XSS. However, it failed to cope with attacks such as DNS Spoofing and Recon-PortScan. The precision was 0.77, the recall was 0.78 and the F1-score was 0.77, which indicates moderate but balanced performance. While CNNs were able to effectively learn simple patterns, they struggled with time-based attacks or others.

**Figure 6:**
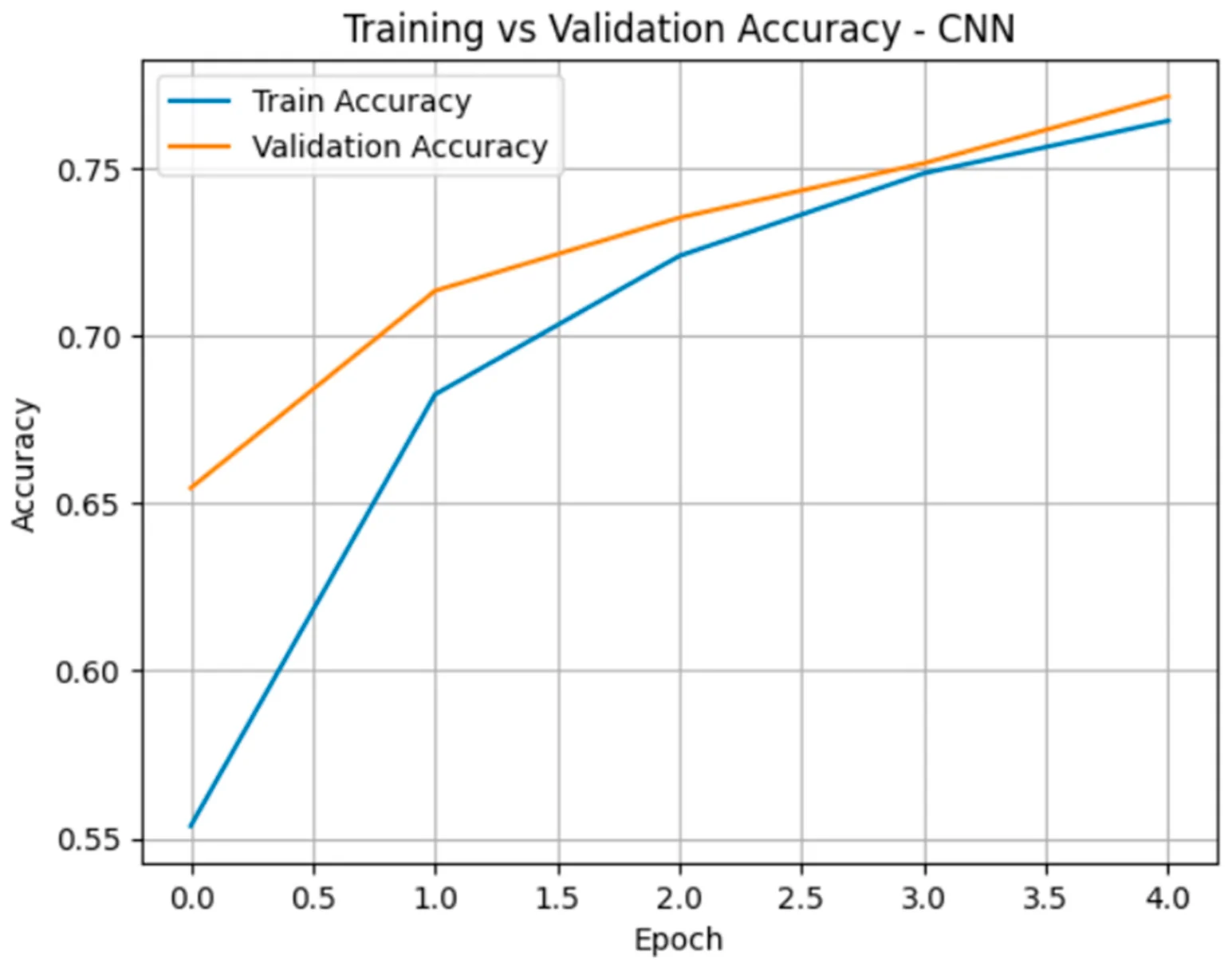
CNN accuracy plot.

Although CNNs are generally used for image data, they performed reasonably with an estimated accuracy of 78%, as shown in Figure 6. The CNN model works for spatial data, but it is not suited for sequential attack data, like attack data from time series, as it lacks the natural ability to learn about time dependencies. But still, it was able to detect many varieties of attacks effectively, owing to its good feature extraction ability.

**Figure 7:**
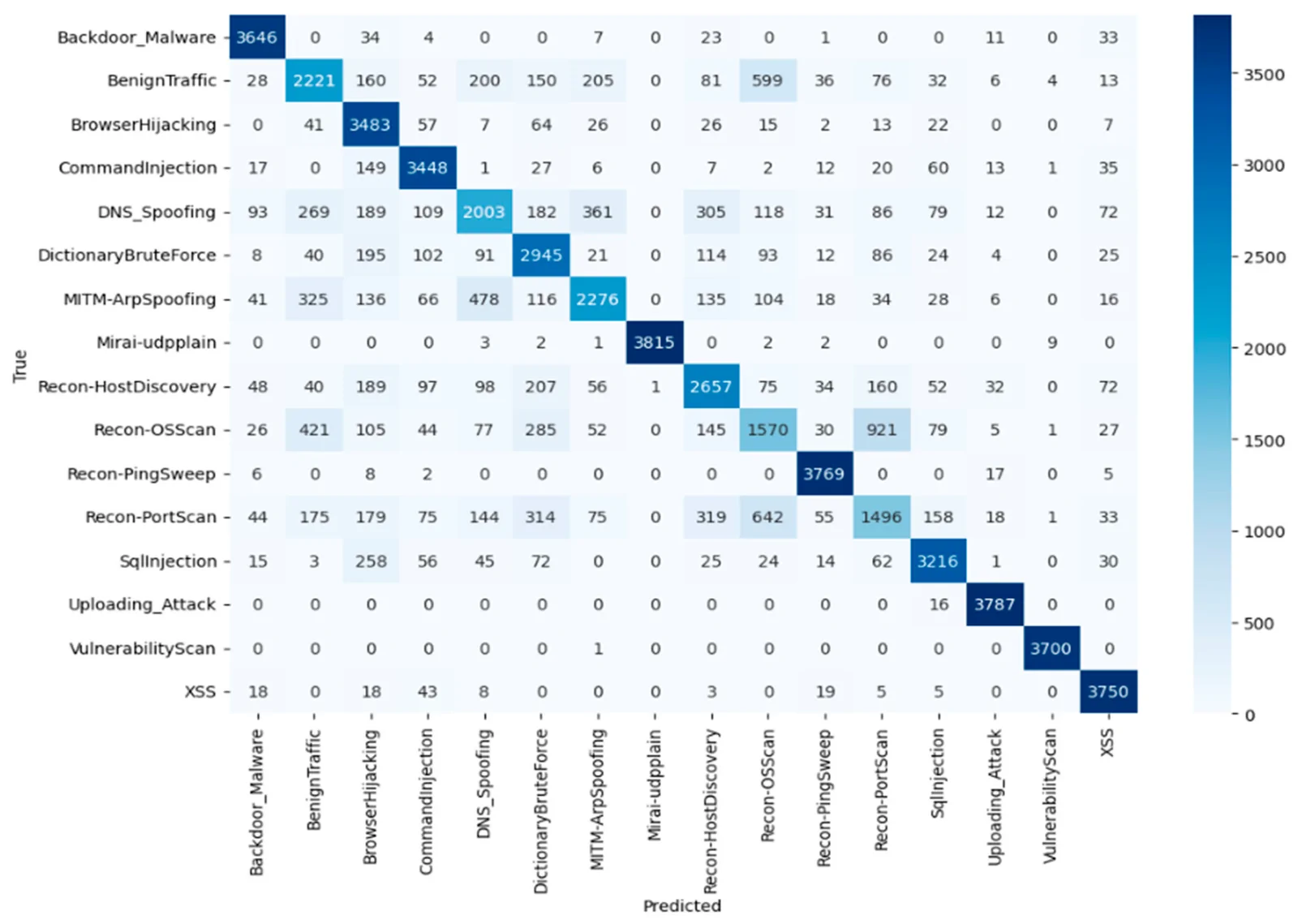
CNN confusion matrix.

The CNN model’s confusion matrix is shown in Figure 7, which indicates its ability to classify all the cyberattacks and regular traffic. Each cell has the following information: actual and anticipated numbers of samples in each class; the darker the color of the cell, the more likely and accurate the model’s prediction was. Based on the data, CNN identified many types of assaults with high accuracy. For instance, it precisely identified BrowserHijacking—3848, Command injection—3448, Uploading Attack—3785, and XSS assaults—3.750. It also performed in other classes, such as Recon-PingSweep and VulnerabilityScan, showing that CNN could detect crucial aspects from various attacks. There are still some misclassifications that can be observed, however. Some were mistakenly identified as attack classes when they were actually benign traffic, for example, and some were misidentified as neighboring classes, such as PortScan for Recon-OSSCan. This indicates that CNN works in spatial pattern recognition, but can miss subtle temporal behavior that is often vital in recognizing more complex or slower moving threats.

#### 4.1.3. DNN

**Figure 8:**
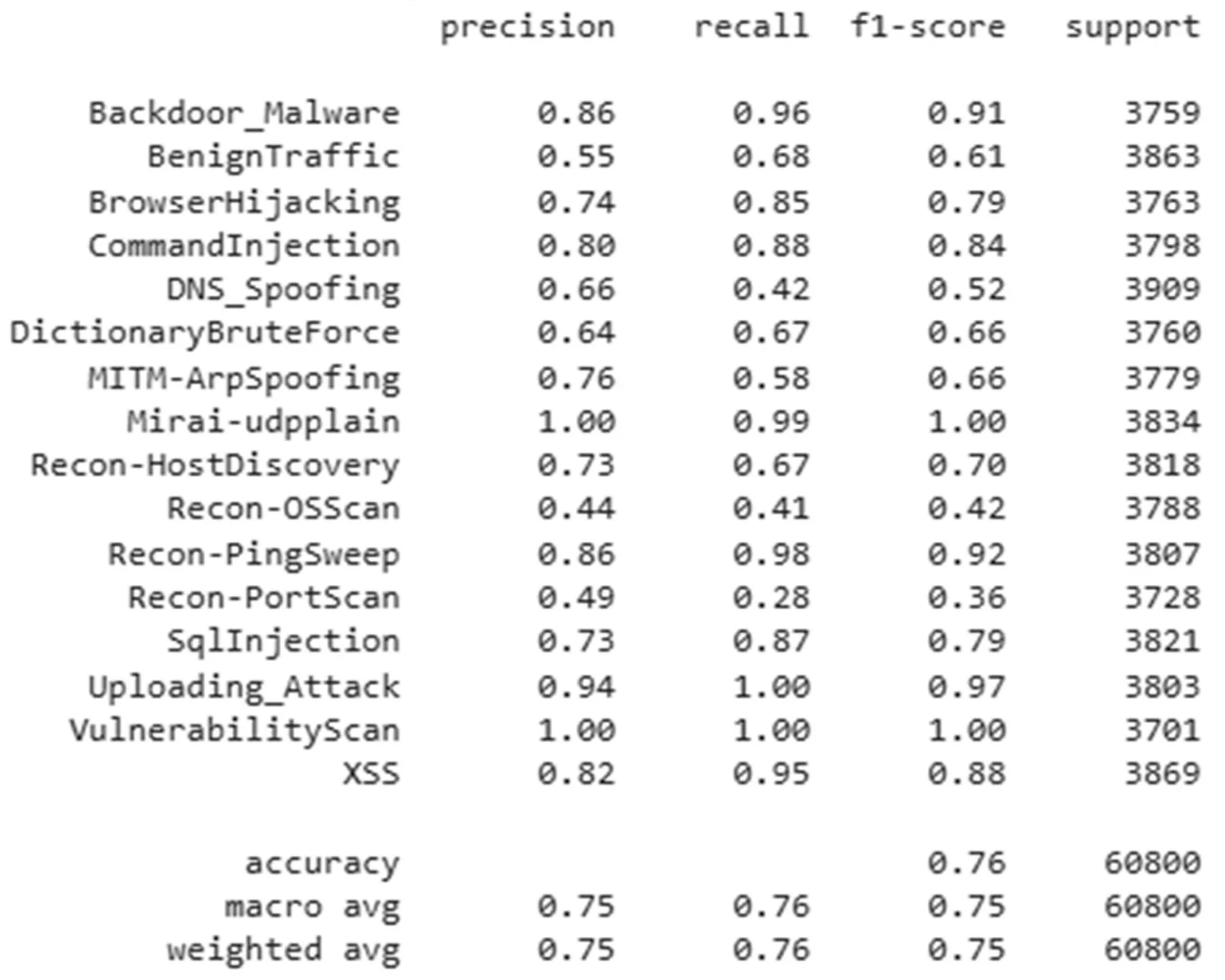
Evaluation metrics for DNN.

As depicted in Figure 8, the DNN model achieved the highest accuracy, 76%, in the Uploading_Attack, VulnerabilityScan and Mirai-UDPPlain tests, with the F1-score being high. For CommandInjection and XSS, the performance was quite satisfactory. Recon-PortScan and DNS Spoofing were problematic, while BenignTraffic only got average results. In general, the detection systems achieved a high specify rate, an average precision of 0.75, a recall of 0.76 and an F1-score of 0.75, except for a few types of attacks.

**Figure 9:**
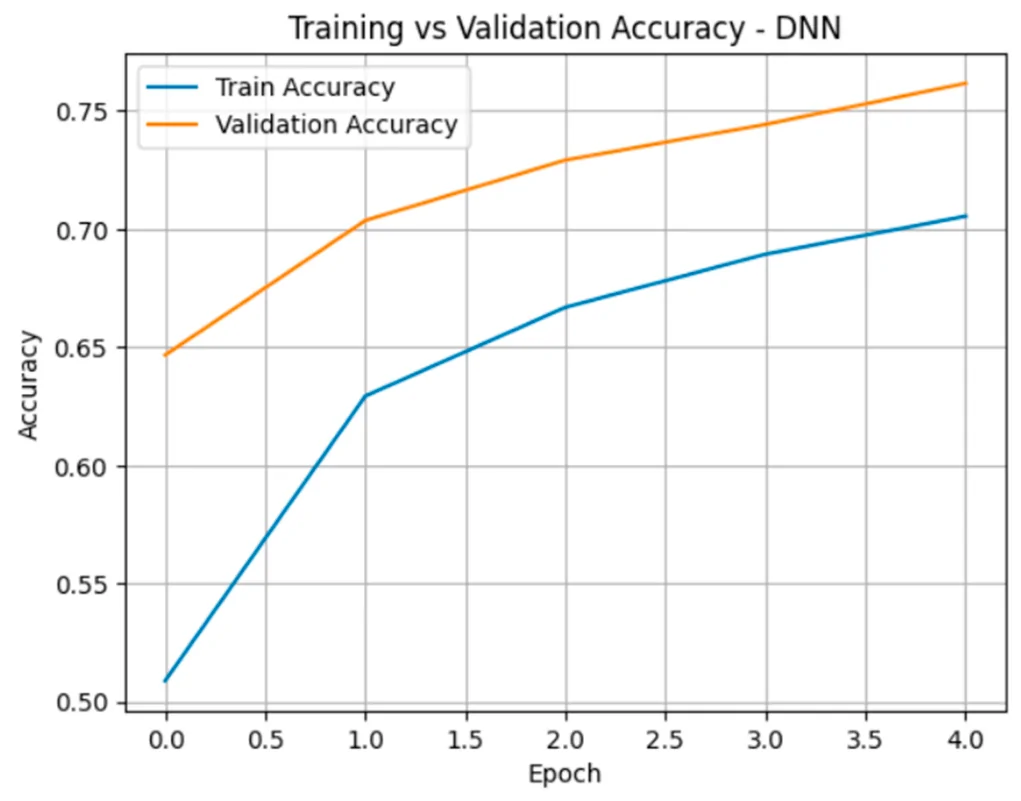
DNN accuracy plot.

The accuracy graph of the DNN model displays an accuracy of approximately 76%, as depicted in Figure 9. It is made up of multiple fully connected layers capable of learning complex patterns in data. However, DNNs are not temporally aware, which restricts their ability to identify time-dependent behavior in network traffic. Nevertheless, they are accurate, easier to train, and serve as a good baseline model.

**Figure 10:**
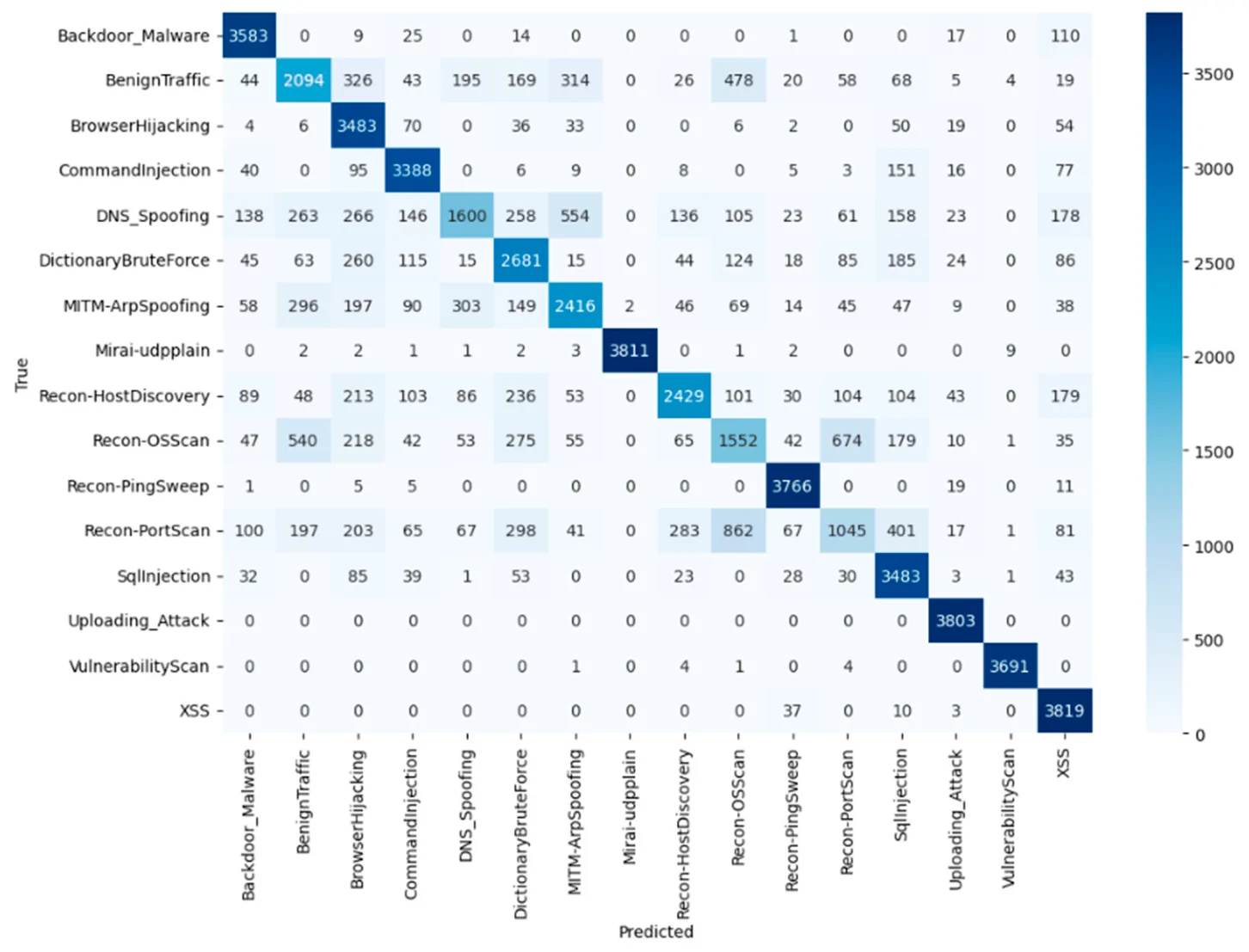
DNN confusion matrix.

As can be seen from the confusion matrix in Figure 10, the DNN model performed well in identifying several types of attacks. It correctly identified a fairly large number of Mirai-UDPPlain (3811 samples), XSS (3819 samples) and Uploading_Attack (3803 samples), and the diagonal values are quite high. Even with these positive results, there are a few erroneous classifications. Slightly confusing for the model were BenignTraffic, which was viewed as either Recon-PingSweep or Recon-OSScan, and DNS_Spoofing, which was viewed as DictionaryBruteForce. As seen in these errors, there seems to be some similarity in attacks, making it more difficult for the DNN model to distinguish. In general, the DNN model is good at detecting the different attacks, but there are some discrepancies in the recognition results that indicate that it can be improved by making further modifications or combining it with other models, particularly to better differentiate between similar attacks.

#### 4.1.4. LSTM

**Figure 11:**
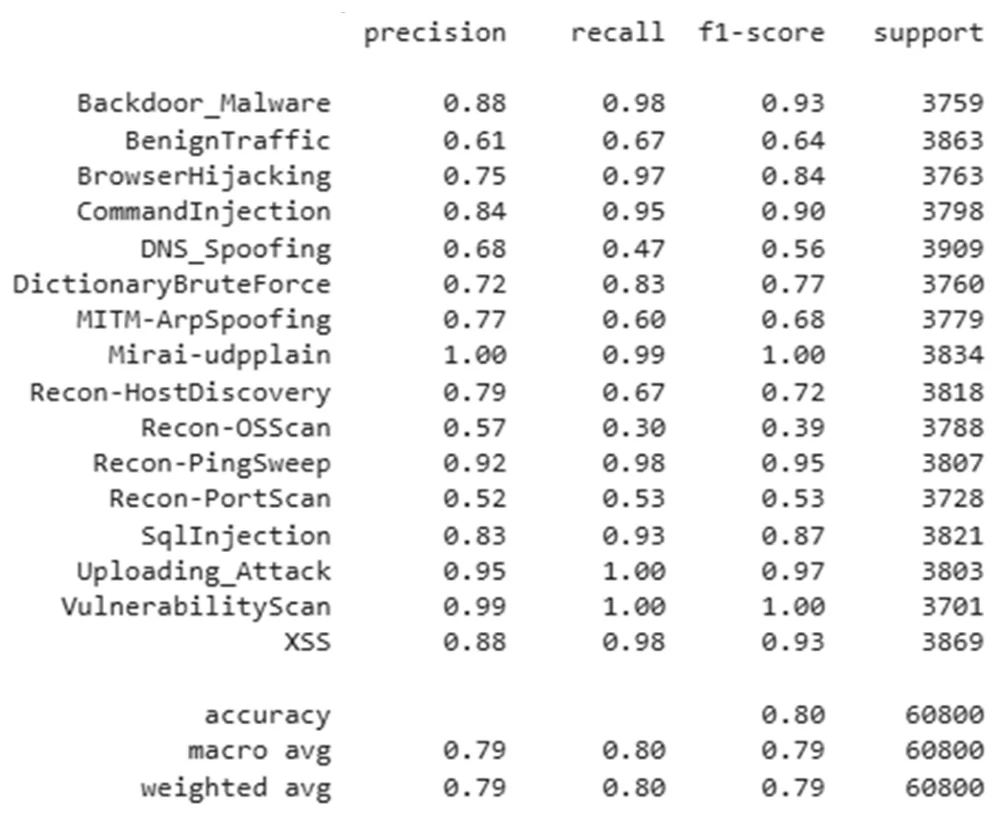
Evaluation metrics for LSTM.

The performance of the LSTM model was the best with 80% accuracy, as shown in Figure 11; this model performed well in most of the attack types. For Mirai-UDPPlain, Uploading_Attack, and VulnerabilityScan, it had perfect, or nearly perfect results. It also protected against CommandInjection, XSS and BrowserHijacking. The overall value of precision was 0.79 and the recall was 0.80, and an F1-score of 0.79 means that the detection was good. It is clear from the above that LSTM was able to learn time-based patterns and outperformed the other models.

**Figure 12:**
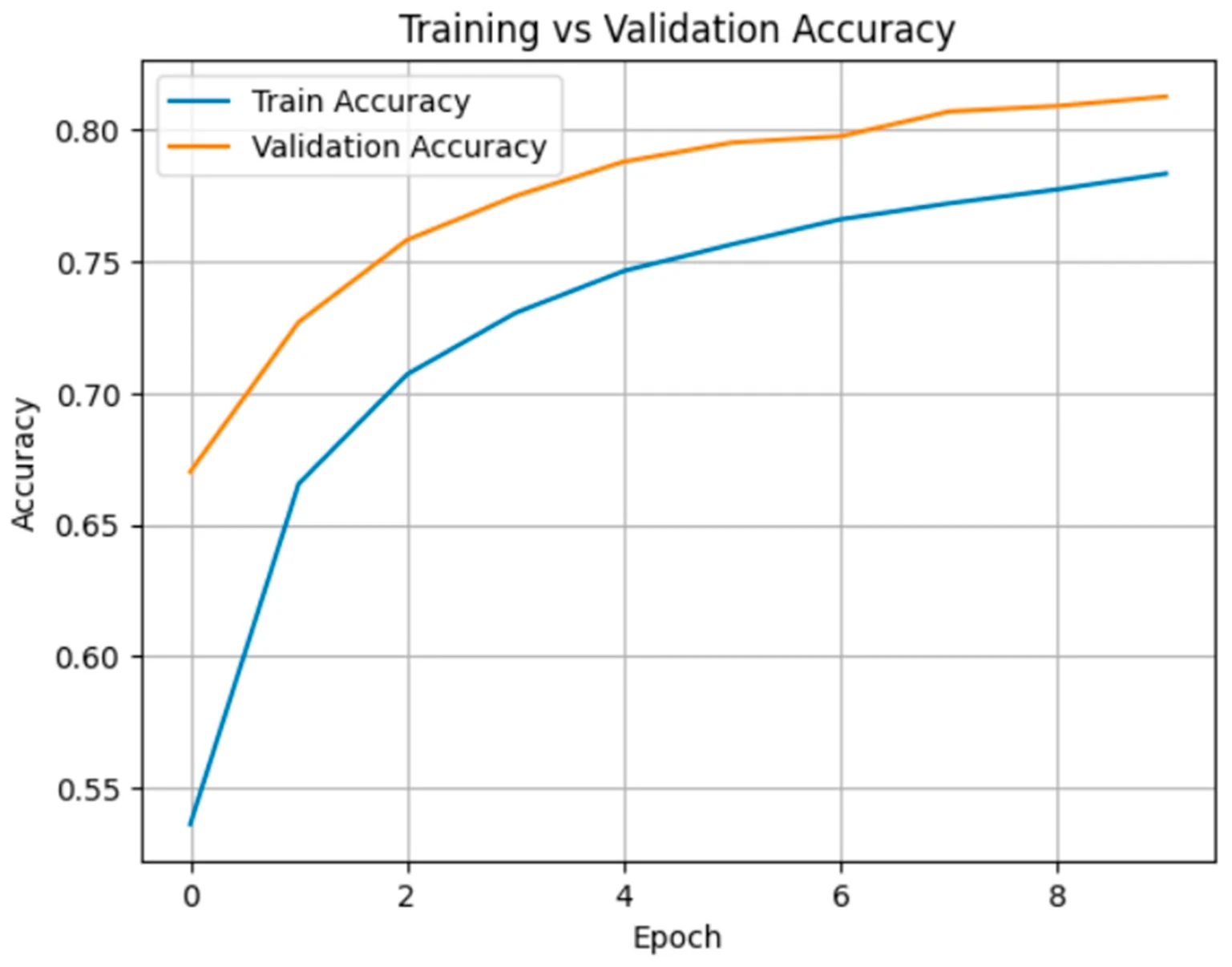
LSTM accuracy plot.

As a variant of the recurrent neural network, LSTM is suitable for time-series network traffic analysis, as it is capable of dealing with long-term dependency in sequential data, achieving 80% accuracy in the graph shown in Figure 12. Its gated structure enables relevant information to be preserved over longer sequences, thus enhancing the ability to detect fast and slow-evolving attack patterns. This helps us towards the target to create an efficient intrusion detection system with the help of a standalone LSTM model.

**Figure 13:**
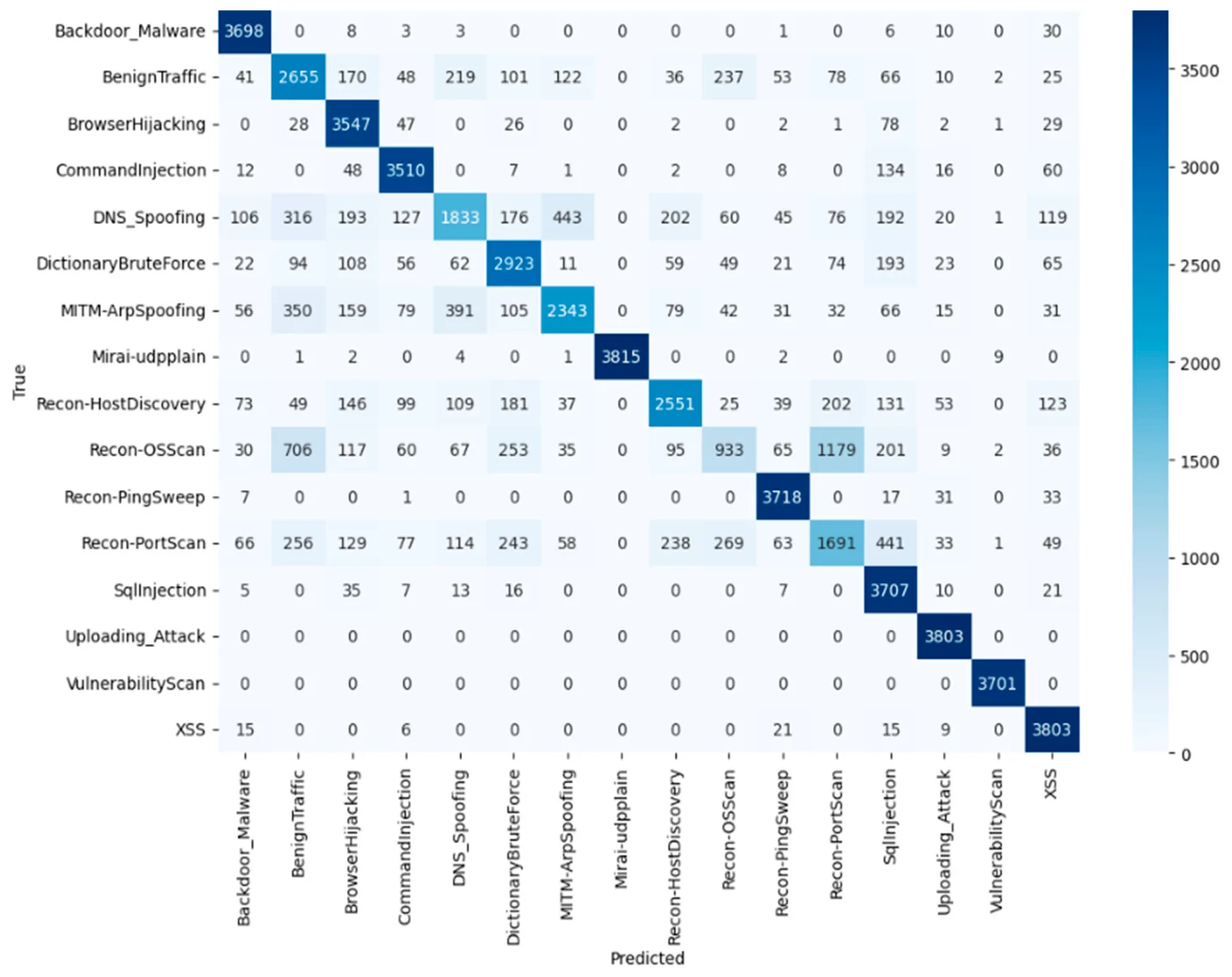
LSTM confusion matrix.

In Figure 13, this confusion matrix shows that the LSTM model performed extremely well in detecting various forms of network attacks and normal traffic. The high counts along the diagonal boxes indicate that it accurately identified most samples across practically all categories. The model performed well in recognizing Mirai-UDPPlain (3815 accurately categorized), XSS (3803), and Uploading_Attack (3803). These findings show that LSTM can accurately detect frequent and modern threats. In addition, the model worked to detect many attack categories, which often share similar patterns. For instance, it had very good performance for Backdoor_Malware (3698) and Command Injection (3510) and did not make any mistakes. Overall, the LSTM model is capable of a variety of threat detection with a high level of accuracy and consistency, which confirms the efficiency of such an approach in the network security domain for intrusion detection. It is capable of complex pattern and sequence recognition, which is desirable for such a security need.

### 4.2. Comparative Analysis

From these comparisons in Table 5, it is obvious that LSTM is a good choice for cyberattack detection in time series network data. Its architecture makes it capable of not only memorizing patterns over time but also adapting to changes in traffic patterns, which is a necessary property in real-world cybersecurity systems. In assuring fair comparison, all models (RNN, CNN, DNN, and LSTM) used the same preprocessing pipeline, including Min–Max normalization, identical train-test splits, identical feature sets, and SMOTE-based balancing applied only to the training subset. Each model was trained and evaluated over ten independent runs with different random seeds. Mean accuracy and standard deviation are illustrated in Table 6.

A paired *t*-test confirmed that the performance improvement of LSTM over CNN was statistically (*p* < 0.05).

**Figure 14:**
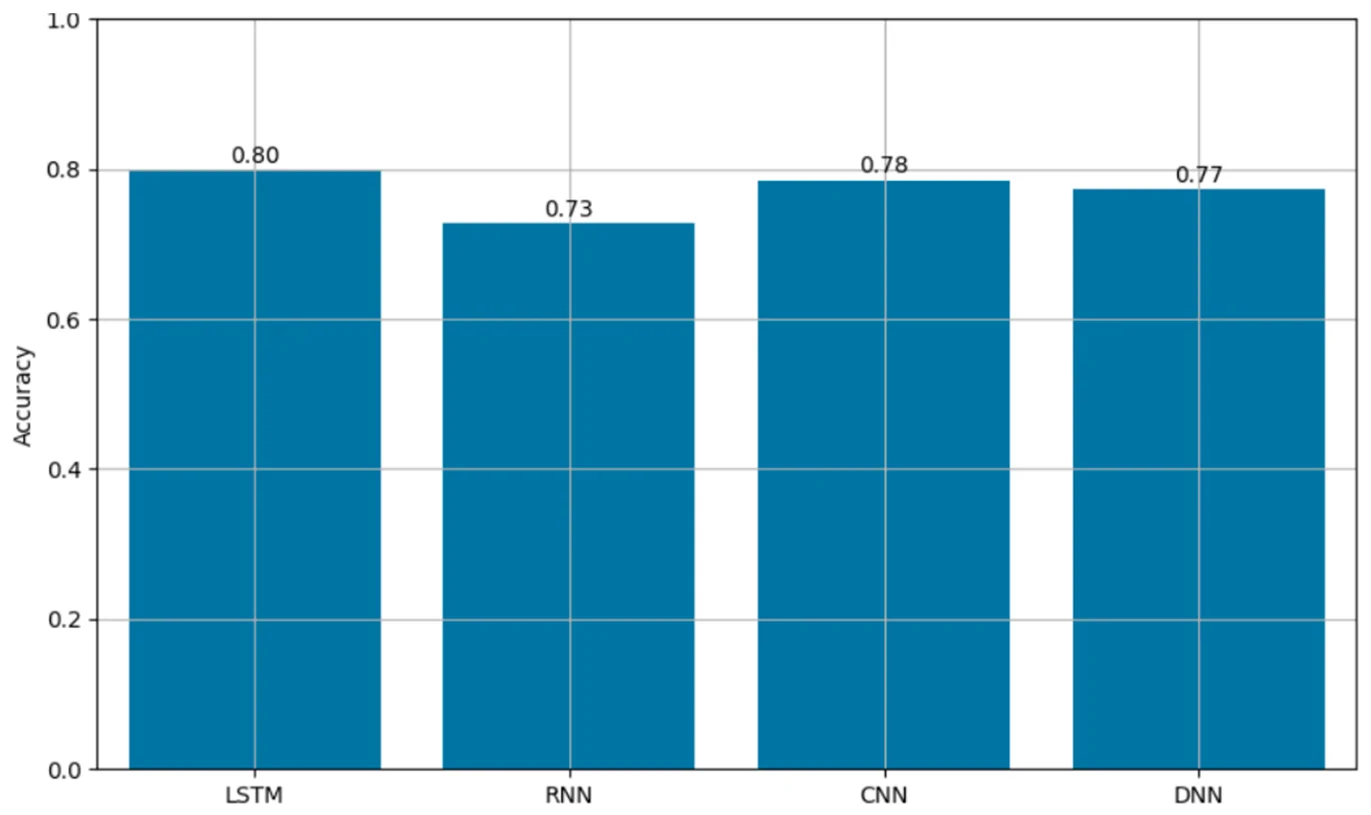
Accuracy comparison of deep learning models for cyber-attack detection.

In Figure 14, the graph clearly shows that LSTM reached a peak of 80%, DNN reached 77%, and CNN reached 78%. For the RNN model, the lowest accuracy was 73%. In identifying cyberattacks, this comparison shows LSTM’s outperformance. The outperformance is specifically apparent within time-based network traffic. It recognizes complex attack patterns through sequential data interpretation, giving it advantages in accuracy and reliability. CNN and DNN did perform well, in general. However, they struggled in detecting the timing of the attacks, which is a critical factor for intrusion detection tasks. In contrast, RNN struggled to retain long-term information, and this resulted in lower performance.

The hyperparameters were determined by empirical testing and common deep learning procedures. A learning rate of 0.001 was used with the Adam optimizer to ensure stable convergence. The choice of a batch size of 64 was designed to optimize the training efficiency and memory utilization, while a dropout rate of 0.2 was used to prevent an overfitting phenomenon. The maximum number of training epochs was also set at 20, as the models always converged after that number of epochs.

To enhance generalization, an early stopping strategy was used. The training data were split into 80% training and 20% validation. The validation loss was tracked after each epoch, and the training was terminated if there was no improvement for five epochs in a row (patience = 5). The model weights that gave the lowest validation loss were loaded for testing.

All experiments were run in the Google Colab environment with an NVIDIA Tesla T4 GPU (12 Gb of VRAM) and an Intel Xeon CPU. The models were written in Python 3.11, TensorFlow 2.16, Keras, NumPy, Pandas, and Scikit-learn. Python, NumPy and TensorFlow were run with a fixed random seed value of 42 to ensure reproducibility.

For each model, we repeated 10 training-and-testing cycles with different random initializations but identical train-test splits. The results presented in Table 4 are the mean accuracy and standard deviations (SD).

## 5. Discussions

The main point emerging from this research is that the success of deep learning models is to a very large extent hinged on their ability to comprehend the sequential nature of behavior in IoT network traffic. The study has unequivocally demonstrated that among the different types of deep learning models, LSTM attained the highest level of performance, being 80% accurate. LSTM is very suitable for detecting cyberattacks that occur temporally, as revealed by this result, which is in line with the known capabilities of LSTM networks that involve memory cells and gating mechanisms to hold the most relevant information for a long time. The LSTM method benefited from IoT traffic in which the attacks were slow-changing or stepwise, and it was therefore able to correctly classify both frequent and rare attacks because of its ability to learn temporal dependencies.

While CNN and DNN were able to achieve quite good accuracy scores of 78% and 76%, respectively, their shortcomings in tightly coupled time-related attack classification became apparent. CNN is characteristically good at spatial pattern recognition, which is a strength in the detection of attacks with distinguished feature patterns; for example, XSS and Command Injection. However, the temporal context of DNS_Spoofing and other Reconnaissance attacks was a challenge to it. Likewise, the DNN model had the potential to uncover the most complex of the feature relationships that it faced, and yet it was unable to handle the progression part of the network activity over time. The models performed in simpler attack scenarios but were less consistent with multi-stage attacks.

Recurrent neural networks (RNNs) had the worst performance, with an accuracy of 73%. One of the major reasons for this was their inability to effectively work with long sequences. The symptom of the vanishing gradient problem was what limited its power—to hold on to the earlier input—while at the same time, it caused it to confuse attack types that were very similar. This evidence supports the notion that ordinarily, RNNs cannot be used as an intrusion detection tool when the tasks involve long sequential patterns.

The use of SMOTE was instrumental in the performance upsurge of the detection of rare attacks, that is, the main point of concern for the focus of this research work. The dataset used for the research was imbalanced in that benign traffic was two or three times more common than the attack samples. Following the implementation of SMOTE, the models—especially LSTM—were able to learn more balanced representations that helped them reduce bias towards the majority classes. This is proof that if data imbalance is not addressed properly in cybersecurity research, the result will be erroneous evaluations.

In general, the outcomes of this research portray the existence of a link between model architecture and attack characteristics. Temporally aware models that retain information best in fast-changing IoT scenarios and time-sensitive deep learning frameworks, such as LSTM, can be very good at uncovering the evolvement of cyberattacks or the fact that they are multi-staged. Although the proposed LSTM model demonstrated promising performance on the CIC_IoT_Dataset2023, additional experiments on datasets such as CICIDS2017, UNSW-NB15, BoT-IoT, and TON_IoT are required to fully assess model generalizability. Future work will evaluate cross-dataset performance and domain adaptation capabilities.

## 6. Conclusions and Future Work

The research presented here is that the Long Short-Term Memory (LSTM) machine learning technique can, when trained on a very diverse range of cyberattacks, find network traffic coming from Internet of Things (IoT) devices that are attacked in widely different ways. By employing a dataset called CIC_IoT_Dataset2023, consisting of up-to-date attacks on the Internet of Things, the proposed LSTM achieved an accuracy of 80%, exceeding the strongest baseline by approximately 2%. It is composed of sequential-based learning, which makes it more feasible to locate attacks that are built up slowly and also incidents that might be multi-step in nature. The LSTM has, to a lesser extent, been able to keep up its eligibility for various occurrences of intrusions, and hence, it could be a more stable method when comparing these four techniques: the convolutional neural network (CNN), the deep neural network (DNN), the recurrent neural network (RNN), and LSTM. In addition to the use of SMOTE, the minority of attack classes had their performances further raised, which is crucial in real-world situations where attacks appear less frequently than normal traffic. The statement that time-dependent cyberattack models are known as far as the security of IoT is concerned is strengthened by these experimental results. The research findings suggest that the LSTM neural network, designed properly, should solve the problem of infection detection, and one should not be in want of intricate hybrid architecture. This is because the model in question attains a very high level of precision, can carry out the same task equally for different data groups, and adapts easily to sequential data. Hence, it is suitable for deployment in real-time systems. Upcoming research may add to this work by exploring model compression to make LSTM more efficient, looking into federated learning to improve privacy levels, and adding explainable AI so that security analysts are facilitated in their decision-making. Thus, LSTM-based IDS models can play a pivotal role in paving the way for a more secure, intelligent, and scalable cybersecurity framework. However, there are a number of limitations to this study. First, experiments were performed using only the CIC_IoT_Dataset2023 dataset. Secondly, the model was tested in an offline environment instead of real deployment in an IoT environment. Thirdly, only deep learning models that were used independently were explored, and other architecture such as hybrid or transformer-based models were not included. Going forward, cross-dataset validation, real-time deployment, and light-weight explainable intrusion detection frameworks will be explored. The proposed methodology is tested with the CIC_IoT_Dataset2023, as it is a recent and comprehensive database of IoT attacks as a benchmark. However, testing the approach on other public datasets like TON_IoT, Bot-IoT, IoT-23 or CICIDS2017 would further prove the generalizability of the model and is intended for future work.

## Figures and Tables

**Table 1:** Class-wise attack details.

Attack Category	Attack Types
**DDoS (12 attacks)**	ACK Fragmentation, HTTP Flood, ICMP Flood, ICMP Fragmentation, PSHACK Flood, RSTFIN Flood, SYN Flood, SlowLoris, Synonymous IP Flood, TCP Flood, UDP Flood, UDP Fragmentation
**DoS (4 attacks)**	HTTP Flood, SYN Flood, TCP Flood, UDP Flood
**Reconnaissance** **(5 attacks)**	Host Discovery, OS Scan, Ping Sweep, Port Scan, Vulnerability Scan
**Web-Based Attacks (6 attacks)**	Browser Hijacking, Command Injection, SQL Injection, Uploading Attack, XSS, Backdoor Malware
**Brute Force (1 attack)**	Dictionary Brute Force
**Spoofing (2 attacks)**	DNS Spoofing, MITM ARP Spoofing
**Mirai (3 attacks)**	Greeth Flood, GreIP Flood, UDPPlain

**Table 2:** SMOTE effect comparison.

Model	Accuracy Without SMOTE	Accuracy with SMOTE
**RNN**	68	73
**CNN**	74	78
**DNN**	71	76
**LSTM**	76	80

**Table 3:** Class-wise attack details.

Attack Category	Attack Class	F1-Score Without SMOTE	F1-Score with SMOTE	Improvement (%)
Benign	Benign	0.98	0.98	0.00
DDoS	UDP Flood	0.91	0.95	+4.40
Web-Based	SQL Injection	0.65	0.79	+21.54
	Uploading Attack	0.61	0.77	+26.23
	XSS	0.68	0.81	+19.12
Mirai	UDPPlain	0.72	0.84	+16.67

**Table 4:** SMOTE effect comparison.

Model	Layers	Units/Filters	Dropout	Activation
**RNN**	1 RNN + Dense	128	0.2	ReLU
**CNN**	Conv1D + MaxPool + Dense	64 filters	0.2	ReLU
**DNN**	Dense-Dense-Dense	256-128-64	0.3	ReLU
**LSTM**	LSTM + Dense	128	0.2	ReLU

**Table 5:** Comparative analysis of deep learning models for cyber attack detection.

Model	Accuracy	Strengths	Weaknesses
RNN	0.73	Handles sequences	Poor long-term memory
CNN	0.78	Detects spatial features	Not designed for sequential data
DNN	0.77	High accuracy with simple structure	Lacks time awareness, weaker with rare attacks
LSTM (Proposed Model)	0.80	Learning long-term, time-dependent patterns	Slower training due to complexity

**Table 6:** Statistical validation.

Model	Mean Accuracy (%)	Std Dev
**RNN**	73.1	0.8
**CNN**	78.2	0.5
**DNN**	76.4	0.6
**LSTM**	80.3	0.4

## Data Availability

The data supporting the findings of this research were obtained from the CIC_IoT_Dataset2023 dataset on Kaggle.
